# Manual of Diseases of the Skin

**Published:** 1886-08

**Authors:** 


2. A Manual of Diseases of the Skin. *By* Balmanno Squire, m. b., *London, Surgeon to the British Hospital for Diseases of the Skin, etc. Chicago:* A. N. Marquis & Co., *Clark and Adams streets. 1886.*

